# Association between mental health status and bone mineral density: Analysis of the 2008-2010 Korea national health and nutrition examination survey

**DOI:** 10.1371/journal.pone.0187425

**Published:** 2017-11-13

**Authors:** Changtae Hahn, Ji Hoon Oh, Soo-Hyun Joo, Jo-Eun Jeong, Jeong-Ho Chae, Chang-Uk Lee, Tae-Suk Kim

**Affiliations:** 1 Department of Psychiatry, Daejeon St. Mary’s Hospital, College of Medicine, Catholic University of Korea, Deajeon, Republic of Korea; 2 Department of Psychiatry, Seoul St. Mary's Hospital, College of Medicine, The Catholic University of Korea, Seoul, Republic of Korea; University of Arkansas for Medical Sciences College of Pharmacy, UNITED STATES

## Abstract

The current study aimed to investigate the association between mental health status and bone mineral density (BMD) using data from the Korean National Health and Nutrition Examination Survey (KNHANES) 2008–2010. We enrolled 15,876 South Korean participants (4,010 postmenopausal females, 4,836 premenopausal females, and 7,016 males, all aged 20 years or older). BMD was measured using dual-energy radiography absorptiometry at the femoral neck (NK), lumbar spine (LSP), and total femur (TFM). Mental health status data were obtained from a self-report questionnaire that assessed psychological stress, depressed mood, and suicidal ideation. Psychological stress was negatively correlated with BMD in the LSP, NK, and TFM for the male group. Depressed mood was associated with lower BMD in the LSP, NK and TFM for the premenopausal female group, and in the LSP for the male group. Suicidal ideation was associated with lower BMD in the NK and TFM for the male group. Mental health problems were associated with lower BMD, especially in premenopausal females and males. Future investigations should focus on the shared pathophysiology between mental health problems and BMD, and the interrelationship between increased BMD and recovery from mental health problems.

## Introduction

In the past decade, mental illnesses have received worldwide attention as a major public health problem.[1] Several investigators reported that mental health problems were significantly associated with poor quality of life,[2] and various mental disorders were related to functional impairment.[3,4] Mental health illnesses are fairly common; approximately 25% of all individuals experience mental health problems at some point in their lifetime.[5] Therefore, patients with physical diseases could have a concurrent mental health disease, whether the two types of disease were independent or interrelated. It is certain that physical and mental disease comorbidity could cause an increased disease burden, not only in terms of effect accumulation on functional impairment severity, but also due to increased health care expenses. Furthermore, from a clinician’s perspective, physical and mental disease comorbidity leads to greater therapeutic challenges and poorer outcomes than if each problem existed independently.[5]

Several investigators found that various mental health problems were significantly associated with physical diseases, particularly chronic conditions, including cardiovascular disease, chronic obstructive pulmonary disease (COPD), and diabetes.[6–8] Osteoporosis is a metabolic bone disease characterized by loss of bone mass and bone tissue microarchitectural deterioration. Osteoporosis is a major health problem that can lead to fragile fractures and increased fracture risk. It is a chronic, prevalent, and debilitating disease that strongly influences morbidity, mortality and quality of life.[9] In this context, several investigators have been concerned that osteoporosis and mental disorders might share a common denominator which indicates the possibility of a mutual relationship. Several studies found that major depressive disorder was associated with lower bone mineral density and that lower bone mineral density in subjects with depression increased fracture risk.[9–12] In addition, some investigators observed that postmenopausal osteoporosis has a specific relationship with depression, [13,14] and several studies explored the shared pathophysiology between osteoporosis and depression.[13,15–20]

In the present study, we aimed to evaluate the association between lower bone mineral density and mental health problems among Korean adults who participated in the Korean National Health and Nutrition Examination Survey (KNHANES, 2008–2010). We hypothesized that mental health problems, including psychological stress and depressive symptoms, as well as lower bone mineral density, would be significantly correlated with one another after controlling for essential demographic data that could present potential risk factors for lower bone mineral density and mental health problems. This study examined relatively extensive demographic data that could identify potential risk factors for lower bone mineral density and/or depression, relative to the above-mentioned studies. Furthermore, we also focused on mental health problems, such as psychological stress and suicidal ideation, as well as depression, the primary focus of previous studies.

## Materials and methods

### Subjects and data collection

The current study analyzed data from the KNHANES (2008–2010), which was a cross-sectional, nationwide, population-based survey conducted by the Division of Chronic Disease Surveillance of the Korean Center for Disease Control and Prevention from 2008 to 2010. The KNHANES is composed of three different surveys on the health and nutrition of the Korean population: a health interview survey, physical examination, and nutrition survey.[21] Health interviews and physical examinations were performed at mobile exam centers, and nutrition data were collected through visits to subject households. The survey acquired a representative national sample from the Korean population using a stratified, multistage, clustered probability sampling design. KNHANES is available to researchers as an open material on the website. (knhanes.cdc.go.kr)

From 2008 to 2010, 29,235 individuals participated in the KNHANES. The present study included 15,876 of the original participants. Among participants whose BMD was measured in the KNHANES (2008–2010), the present study included only those aged 20 years or older. We then divided participants into three groups: males, premenopausal females, and postmenopausal females. Since menopause has been related to decreased bone mineral density at various skeletal sites and men do not undergo menopause, our three-group classification sought to clarify the substantive correlation between bone mineral density and mental health while simultaneously considering hormonal changes in females and gender differences.

We excluded subjects with missing data on essential analytic variables, such as bone mineral density (BMD) and the mental health questionnaire. The Institutional Review Board of the Centers for Disease Control and Prevention in Korea approved use of KNHANES (2008–2010) data. All participants of the KNHANES used in the current study provided written informed consent.

### Bone mineral density measurement

A trained radiographer in a mobile examination center using dual-energy radiography absorptiometry (DXA; DISCOVERY-W fan-beam densitometer; Hologic, Bedford, MA, USA) measured BMD at the femoral neck, lumbar spine, and total femur. This device used Hologic Discovery software, version 3.1. Accuracy was evaluated by performing double scans on 30 random samples. Coefficients of variation (CVs) for accurate BMD assessment were 1.9% for the lumbar spine, 2.5% for the femur neck, and 1.8% for the total femur. Calibration was performed daily. We analyzed lumbar spine, femur neck, and total femur BMD.

### Mental health measures

Mental health data were obtained from a self-report mental health questionnaire administered under the supervision of a researcher. Mental health surveys were divided into questions from three domains: stress, depression, and suicidal ideation and attempts. Stress level stress was assessed by asking, "What are your stress levels?” Possible responses included none, mild, moderate, or severe. We divided research participants into two groups based on reported stress levels (none to mild and moderate to severe). Depressive symptoms were assessed by asking, "Have you felt any sadness or hopelessness that has interfered with your daily life for two or more continuous weeks during the past year?" Participants were placed into a dichotomous category according to their answer, "yes" or "no." Watkins and colleagues evaluated the validity of this indicator for depressive symptoms. [22] Suicidal ideation was assessed by asking, "Did you ever think about committing suicide?" Participants were divided into two groups according to their answer, "yes" or "no." Gaynes and colleagues reported that this assessment method was a useful predictor of suicidal attempts in a survey of suicidal risk. [23]

### Statistical analysis

We presented data as mean ± standard error (SE) for continuous variables or as percentages (SE) for categorical variables. Analysis of variance was used for continuous variables, and a Chi-square test was applied to categorical variables to quantify differences in participant characteristics. A multi-variable logistic regression model was employed to analyze the relationship between mental health measures and BMD for every increment of 0.1 g/cm^2^. Model 1 was adjusted for age and body mass index (BMI). Model 2 was adjusted for model 1 covariates, as well as cigarette smoking, alcohol intake, regular exercise, education and income. Model 3 was adjusted for model 2 covariates, as well as metabolic syndrome, diabetes mellitus (DM), and hypertension (HTN). Analysis of covariance was used to compare BMD between postmenopausal, premenopausal and male groups covaried according to the three mental health domains of stress, depression, and suicidal ideation. Stress level was organized into two groups (none and mild vs moderate and severe). Depressive symptoms and suicidal ideation were organized into dichotomous categories (yes or no). The Statistical Analysis System (SAS) software package version 9.2 for Windows (SAS Institute Inc., Cary, NC, USA) was used to accommodate the complex sampling design and weighting inherent in the KNHANES, and to provide approximations for the entire Korean population. All statistical tests were two-tailed, and a *p* < 0.05 was considered statistically significant.

## Results

Table 1 presents study population sociodemographic characteristics (postmenopausal female, premenopausal female, and male groups). Mean age was 62.8±0.2 years for postmenopausal females, 35.3±0.2 years for premenopausal females, and 43.7±0.3 years for males. Table 1 shows BMD values for the three groups (lumbar spine (LSP), total femur (TFM), and femoral neck (NK)). Table 1 shows body mass index (BMI), waist circumference, sleep duration, smoking, drinking, exercise, education, income, residence, metabolic syndrome, DM, and HTN for the three study groups. Table 1 contains information about covariates that should be considered.

**Table 1:** General participant characteristics.

	Postmenopausal females (N = 4,010)	Premenopausal females (N = 4,836)	Males (N = 7,016)
Age (years)	62.8(36.0/64.1/95.0, 0.2)	35.3(20.0/37.0/59.0, 0.2)	43.7(20.0/49.0/93.0, 0.3)
BMI (kg/m^2^)	24.2(14.0/24.0/42.1, 0.1)	22.5(13.7/22.1/46.2, 0.1)	24(0.0)
Waist circumference (cm)	82.2(54.5/82.1/121.9, 0.2)	74.9(52.1/74.2/127.0, 0.2)	83.9(36.2/84.2/166.1, 0.2)
Sleep duration (hours)	6.5(1.0/7.0/14.0, 0.0)	7.1(1.0/7.0/15.0, 0.0)	6.9(1.0/7.0/14.0, 0.0)
DX_LSP_BMD (g/cm^2^)	0.808(0.327/0.788/1.577, 0.003)	0.984(0.608/0.983/1.495, 0.002)	0.974(0.294/0.957/1.550, 0.002)
DX_TFM_BMD (g/cm^2^)	0.776(0.352/0.766/1.225, 0.002)	0.901(0.589/0.900/1.300, 0.002)	0.979(0.431/0.956/1.487, 0.002)
DX_NK_BMD (g/cm^2^)	0.625(0.238/0.613/1.097, 0.002)	0.765(0.463/0.758/1.202, 0.002)	0.828(0.296/0.789/1.413, 0.002)
Ever-smoker (%)	9.0(0.6)	12.3(0.6)	77.4(0.6)
Drinker—heavy (%)	47.5(1.1)	77.8(0.8)	86.5(0.5)
Regular exerciser (%)	22.0(0.9)	22.9(0.8)	27.9(0.7)
Education—≥high school (%)	21.9(1.0)	88.9(0.6)	77.1(0.7)
Income—lower quartile (%)	33.6(1.1)	9.2(0.6)	14.4(0.6)
Residence—urban (%)	71.7(2.3)	85.3(1.6)	79.9(1.8)
Metabolic syndrome (%)	55.1(1.2)	13.2(0.7)	28.9(0.9)
Diabetes Mellitus (%)	14.9(0.7)	2.7(0.3)	8.9(0.4)
Hypertension (%)	50.5(1.0)	7.8(0.4)	31.6(0.8)

BMD, bone mineral density; BMI, body mass index; DX, dual-energy radiography absorptiometry; LSP, lumbar spine; NK, femoral neck; TFM, total femur

Ever-smoker: past and present smoker (at least 100 cigarettes)

Drinker—heavy: average 30g/day or more alcohol consumption

Regular exercise: strenuous physical activity performed for at least 20 min at a time at least three times a week

Data were shown as mean (standard error) or percentage (standard error).

Continuous variables were shown as mean (minimum/median/maximum, standard error).

Table 2 presents differences in mental health status between the postmenopausal female, premenopausal female and male groups. The proportion of postmenopausal females, premenopausal females, and males who reported moderate to severe stress was 29±0.9%, 34±0.8%, and 27.1±0.7% (p = 0.0001), respectively. Depressed mood for two or more continuous weeks was reported by 22.1±0.8% of postmenopausal females, 16.4±0.6% of premenopausal females, and 9.2±0.4% of males (p < .0001). Suicidal ideation was reported by 27.4±1% of postmenopausal females, 18.5±0.7% of premenopausal females, and 10.6±0.4% of males (p < .0001).

**Table 2:** Participants with positive responses to mental health questionnaires.

	Females		Males	*p* value
	Postmenopausal	Premenopausal		
Stress (moderate to severe)	29.0(0.9)	34.0(0.8)	27.1(0.7)	< .0001
Depressed mood	22.1(0.8)	16.4(0.6)	9.2(0.4)	< .0001
Suicide ideation	27.4(1.0)	18.5(0.7)	10.6(0.4)	< .0001

Data were shown as percentage (standard error).

Analysis of covariance (ANCOVA) was used to compare BMD between postmenopausal, premenopausal and male groups covaried according to the three mental health domains of stress, depression, and suicidal ideation. And the results of ANCOVA showed statistically significant differences. Table 3 presents results from the multivariate logistic regressions analyzing the relationship between BMD and mental health among postmenopausal females, premenopausal females, and males. Moderate to severe stress was correlated with decreased BMD at the LSP, NK, and TFM in the male group after adjusting for age and BMI (Model 1). For the male group, after adjusting for age and BMI, smoking, drinking, exercise, education and income (Model 2), the correlation between moderate to severe stress and decreased BMD remained significant, as well as after adjusting for age and BMI, smoking, drinking, exercise, education, income, metabolic syndrome, DM and HTN (Model 3). These covariates have been linked to BMD in previous studies analyzing KNHANES data. [24–26] Through the stepwise analysis (Model 1, 2, 3), we considered the importance of selecting covariates which was controversial and difficult tasks. Depressed mood was associated with lower BMD at the LSP and NK among premenopausal females in Models 1 and 2. In Model 3, depressed mood showed a negative correlation with BMD at the LSP, NK and TFM in premenopausal females, and at the LSP for males. Suicidal ideation was related to lower BMD at the TFM in postmenopausal females, at the LSP and NK in premenopausal females, and at the NK and TFM in males in Model 1. In Model 2, suicidal ideation was associated with lower BMD at the LSP and NK in premenopausal females and at the NK and TFM in males. In Model 3, suicidal ideation was correlated with lower BMD at the NK and TFM in males.

**Table 3:** The relationship between mental health status and bone mineral density of postmenopausal females, premenopausal females and males.

		Postmenopausal females		Premenopausal females		Males	
		OR(95% CI)	*p* value	OR(95% CI)	*p* value	OR(95% CI)	*p* value
Stress (moderate to severe)							
**M** **O** **D** **E** **L** **1**	LSP	1.05(0.98,1.12)	0.19	0.98(0.91,1.04)	0.48	0.93(0.88,0.97)	**< .001**
	NK	0.94(0.86,1.03)	0.19	0.96(0.89,1.03)	0.21	0.89(0.84,0.95)	**< .001**
	TFM	0.96(0.88,1.04)	0.30	0.98(0.91,1.06)	0.62	0.91(0.86,0.96)	**< .001**
**M** **O** **D** **E** **L** **2**	LSP	1.05(0.98,1.13)	0.14	0.98(0.92,1.05)	0.56	0.93(0.88,0.98)	**< .001**
	NK	0.96(0.87,1.05)	0.33	0.96(0.89,1.03)	0.25	0.90(0.85,0.95)	**< .001**
	TFM	0.97(0.89,1.06)	0.51	0.99(0.92,1.07)	0.83	0.91(0.86,0.97)	**< .001**
**M** **O** **D** **E** **L** **3**	LSP	1.06(0.96,1.16)	0.26	0.94(0.86,1.01)	0.09	0.90(0.84,0.96)	**< .001**
	NK	0.99(0.88,1.11)	0.82	0.95(0.88,1.03)	0.24	0.82(0.76,0.88)	**< .001**
	TFM	1.00(0.89,1.12)	0.93	0.97(0.89,1.06)	0.47	0.86(0.80,0.92)	**< .001**
Depressed mood							
**M** **O** **D** **E** **L** **1**	LSP	0.99(0.91,1.06)	0.69	0.89(0.82,0.97)	**0.01**	0.94(0.87,1.01)	0.11
	NK	0.92(0.82,1.02)	0.11	0.90(0.82,0.98)	**0.02**	0.93(0.85,1.03)	0.16
	TFM	0.93(0.84,1.04)	0.20	0.93(0.85,1.02)	0.12	0.93(0.85,1.02)	0.14
**M** **O** **D** **E** **L** **2**	LSP	1.01(0.93,1.10)	0.76	0.91(0.83,0.99)	**0.02**	0.97(0.90,1.05)	0.41
	NK	0.93(0.84,1.05)	0.23	0.90(0.82,0.99)	**0.02**	0.97(0.88,1.06)	0.47
	TFM	0.95(0.85,1.06)	0.33	0.94(0.85,1.03)	0.15	0.97(0.89,1.07)	0.58
**M** **O** **D** **E** **L** **3**	LSP	0.98(0.88,1.09)	0.64	0.90(0.81,0.99)	**0.04**	0.90(0.82,0.99)	**0.04**
	NK	0.88(0.77,1.01)	0.07	0.83(0.74,0.93)	**< .001**	0.91(0.80,1.03)	0.13
	TFM	0.90(0.78,1.03)	0.13	0.86(0.78,0.96)	**0.01**	0.92(0.82,1.03)	0.15
Suicidal ideation							
**M** **O** **D** **E** **L** **1**	LSP	0.98(0.91,1.05)	0.49	0.91(0.84,0.98)	**0.01**	0.94(0.87,1.00)	0.07
	NK	0.90(0.80,1.00)	0.05	0.90(0.83,0.98)	**0.02**	0.87(0.79,0.95)	**< .001**
	TFM	0.90(0.82,0.10)	**0.04**	0.92(0.85,1.00)	0.06	0.86(0.79,0.94)	**< .001**
**M** **O** **D** **E** **L** **2**	LSP	1.01(0.94,1.09)	0.79	0.92(0.86,0.99)	**0.03**	0.96(0.90,1.03)	0.27
	NK	0.94(0.84,1.05)	0.29	0.91(0.83,0.99)	**0.02**	0.89(0.81,0.98)	**0.02**
	TFM	0.94(0.85,1.04)	0.24	0.94(0.86,1.02)	0.12	0.89(0.81,0.97)	**0.01**
**M** **O** **D** **E** **L** **3**	LSP	1.02(0.93,1.12)	0.65	0.94(0.86,1.03)	0.18	0.96(0.88,1.04)	0.30
	NK	0.95(0.83,1.09)	0.48	0.93(0.84,1.03)	0.15	0.86(0.76,0.97)	**0.02**
	TFM	0.95(0.84,1.07)	0.39	0.96(0.87,1.06)	0.44	0.88(0.79,0.98)	**0.02**

MODEL 1: Adjusted for age and BMI.

MODEL 2: Adjusted for age and BMI, smoking, drinking, exercise, education and income.

MODEL 3: Adjusted for age and BMI, smoking, drinking, exercise, education, income, metabolic syndrome, DM and HTN.

OR = odds ratio; CI = confidence interval; LSP = lumbar spine; TFM = total femur; NK = femoral neck

## Discussion

Several previous studies have demonstrated the relationship between BMD and mental health. [9–20] However, these studies included a relatively small number of participants, and they concentrated primarily on depression, [9–12,27,28] although Guo and colleagues reported an association between osteoporosis and various psychiatric disorders (depression, bipolar disorder, schizophrenia, and Alzheimer's disease).[29] Thus, our study intended to explore the relationship between BMD and various mental health problems (stress, depressive symptoms and suicidal ideation). Furthermore, we considered the level of stress that individuals experienced, even if it was a subclinical state. In addition, we used representative data from KNHANES conducted from 2008 to 2010, which allowed us to include a large number of participants.

This study’s findings indicated that various psychological symptoms were associated with BMD at three sites (LSP, NK, and TFM) in postmenopausal females, premenopausal females and males.

We observed that moderate to severe psychological stress was associated with lower BMD at the LSP, NK and TFM in the male group, while there was no correlation between stress and BMD in postmenopausal or premenopausal females (Table 3). Psychological stress can lead to alterations in the secretion of many hormones, including cortisol, catecholamines, vasopressin, gonadotropins, thyroid hormones, growth hormone, prolactin and insulin.[30] Furthermore, several investigators have suggested that psychological stress could result in decreased testosterone secretion. [17,18] The hypothalamic-pituitary-adrenal axis, which is influenced by psychological stress, could effect inhibitory action on the hypothalamic-pituitary-gonadal (HPG) axis. Inhibition of the HPG axis results in decreased testosterone secretion.[31] A lower serum testosterone concentration is associated with decreased BMD, resulting in osteoporosis. [32] Our findings were consistent with the studies described above. Furthermore, our observation of the association between psychological stress and BMD after adjusting for age and BMI, smoking, drinking, exercise, education, income, metabolic syndrome, DM and HTN (Model 3) suggests a primary role for biological factors, including hormonal alterations.

Depressed mood was associated with lower BMD at the LSP and NK in premenopausal females in Models 1 and 2. In Model 3, depressed mood was correlated with decreased BMD at the LSP, NK and TFM in premenopausal females, and at the LSP in males (Table 3). Depressed mood was also closely associated with BMD in the premenopausal female group, although the BMD at the LSP in the male group was related to depressed mood. Although stress could affect biological alterations, including decreased female sex hormones in women [33], our findings indicate that stress, which might be subjective or subclinical, had little influence on BMD alteration in the female groups. However, depressed mood, which can be more severe than subjective or subclinical stress, can cause biological alterations, including hormonal changes. Considerable psychological burdens, like depressed mood, could influence the abundant state of female sex hormones and lead to biological alterations, including decreased estrogen, which results in lower BMD. Depressed mood was not associated with BMD in the postmenopausal female group, although it was in the premenopausal female group. The reason lies in that dramatic hormonal changes over a woman’s life course, such as menopause, could have a greater influence on BMD than psychological changes, like depressed mood. Furthermore, notably lower estrogen levels in the postmenopausal female group could make these women less sensitive to depressed mood. Therefore, our findings indicate that the postmenopausal female and male groups had similar patterns in the association between depressed mood and BMD alteration. In addition, we could hypothesize that the alteration of male sex hormones was less sensitive than it of female sex hormones to the depressed mood at least. It is interesting that subjective or subclinical stress was closely associated with lower BMD in the male group, unlike the association between BMD and depressed mood.

Suicidal ideation, one of the most severe mental health problems, was related to lower BMD at the TFM in postmenopausal females, at the LSP and NK in premenopausal females, and at the NK and TFM in males in Model 1. It might be that suicidal ideation affects female and male sex hormonal changes and decreased BMD in spite of lower hormonal levels, like in the case of postmenopausal females. However, in Model 2, suicidal ideation was associated with lower BMD at the LSP and NK in premenopausal females and at the NK and TFM in males. In Model 3, suicidal ideation was correlated with lower BMD at the NK and TFM in males. Several investigators found that there were gender differences in the effects of various demographic and risk factors for suicidal ideation.[34–36] Indeed, in Model 3, which was adjusted for age, BMI, smoking, drinking, exercise, education, income, metabolic syndrome, DM and HTN, suicidal ideation was correlated with lower BMD in males. Suicidal ideation could be the cumulative product of various mental health problems, although stress and depressed mood were relatively singular and homogenous expressions of mental health problems. Such a severe mental health problem could be correlated with lower BMD in males and females (Model 1). However, our findings showed that menopause could have a significant impact on lower BMD and that severe psychopathology, such as suicidal ideation, was not significantly associated with decreased BMD (Model 2). Furthermore, suicidal ideation, a severe psychopathology, was correlated with lower BMD in males compared to females. (Model 3)

This study has several limitations. First, this study investigated individuals from only one Asian country, although we analyzed a comparatively large sample. Second, this cross-sectional study was not able to establish causation between mental health and BMD. Third, there was no information about whether participants received psychiatric treatment, including psychiatric medications. Fourth, we could not determine if decreased BMD, which was associated with mental health problems, represented a clinically significant decrease, such as osteomalacia or osteoporosis. Fifth, we investigated the relationship between mental health status and BMD among three groups (postmenopausal females, premenopausal females and males). Such a design might be advantageous in terms of considering menopause. But women have been divided into two categories according to pre- and postmenopausal with a discrepancy of mean age in-between the two female groups although we adjusted for age statistically. Sixth, KNHANES mental health data were composed of self-report scale. Therefor we should considered limitations in terms of their reliability and validity. Lastly, we did not assess male and female sex hormone levels, such as estrogen and testosterone. Therefore, additional longitudinal studies regarding various mental health problems and BMD should be conducted in other races or countries. Furthermore, follow-up studies should include various medical examinations and histories.

In conclusion, evidence from this study indicates that mental health problems were associated with lower BMD, especially among premenopausal females and males. Future investigations should focus on the shared pathophysiology between mental health problems and BMD, and the interrelationship between increased BMD and recovery from mental health problems.

## Supporting information

S1 Data (XLSX)
